# Simultaneous implant placement with guided bone regeneration for horizontal ridge augmentation using a 3D-preformed resorbable PLGA membrane: A prospective single-arm clinical study

**DOI:** 10.1007/s00784-026-06959-9

**Published:** 2026-06-04

**Authors:** Sang-Hyun Son, Kyeong-Ok Lim, Seo-Hee Han, Gwi-Hyeon Min, Jooseong Kim, Tadeusz Morawiec, Won-Pyo Lee

**Affiliations:** 1 Department of Periodontology, School of Dentistry, Chosun University, Gwangju, Republic of Korea; 2 Periodontal R&D Center, Osstem Implant Co., LTD., Seoul, Republic of Korea; 3 Saramsarang Dental Clinic, Pyeongtaek, Republic of Korea; 4 Osong Medical Innovation Foundation, Osong, Republic of Korea; 5 Department of Oral Surgery, Faculty of Medical Sciences in Zabrze, Medical University of Silesia, Katowice, Poland; 6 School of Computer Science, University of Birmingham, Birmingham, UK

**Keywords:** Alveolar bone loss, Biocompatible materials, Bone regeneration, Dental implants, Polylactic acid-polyglycolic acid copolymer

## Abstract

**Objectives:**

This study aimed to evaluate the clinical effectiveness and safety of guided bone regeneration (GBR) using a three-dimensional preformed resorbable Polylactic-co-glycolic acid membrane (3D-PRPM) with simultaneous implant placement.

**Materials and methods:**

Twenty patients (21 sites) with localized ridge defects were treated using implants placed concurrently with GBR using a 3D-PRPM. Cone-beam computed tomography scans were taken preoperatively, immediately postoperatively, and at 5 months. Horizontal bone augmentation (BA), hard tissue gain (HG), bone resorption (BR), and hard tissue gain rate (HGR) were assessed. Changes beyond the original bony envelope were evaluated. Statistical analyses included the Wilcoxon signed-rank and Friedman tests.

**Results:**

Mean BA was 2.99 ± 1.15 mm, and mean HG at 5 months was 2.64 ± 1.10 mm. Mean BR was 0.35 ± 0.23 mm, and mean HGR was 87.2% ± 8.2%. Envelope analysis confirmed a significant increase after augmentation with partial reduction during healing; however, ridge dimensions at 5 months remained significantly greater than baseline (*P* < 0.001). No membrane exposure, infection, or wound dehiscence occurred.

**Conclusion:**

3D-PRPM enabled predictable horizontal ridge augmentation with excellent volumetric stability and favorable short-term safety.

**Clinical relevance:**

A 3D-PRPM may provide a stable, fixation-free resorbable barrier for horizontal ridge augmentation, potentially simplifying GBR procedures and reducing the need for secondary removal.

## Introduction

Insufficient alveolar ridge volume remains one of the most critical challenges for successful implant placement. Guided bone regeneration (GBR) is a well-established surgical approach to overcome this limitation by excluding non-osteogenic tissues and ensuring space maintenance for new bone formation [1, 2]. Central to this technique is the barrier membrane, broadly classified as either non-resorbable or resorbable.

Non-resorbable membranes, particularly titanium meshes, provide excellent stability, space maintenance, and highly predictable outcomes [3, 4]. Nevertheless, their clinical use is limited by frequent exposure, reported in 15–25% of cases and up to 40–50% in some studies [5–8]. Such exposure may result in infection, wound dehiscence, or graft loss, and secondary removal is often necessary, increasing both patient morbidity and surgical complexity [9, 10].

Resorbable collagen membranes were introduced to overcome these drawbacks. While they offer favorable biocompatibility and ease of handling, their low rigidity predisposes them to collapse in large or non-contained defects, often leading to loss of space and compromised outcomes [11]. These limitations underscore the need for resorbable membranes that combine mechanical stability with the advantage of spontaneous degradation, eliminating the requirement for removal.

Polylactic-co-glycolic acid (PLGA) is a synthetic copolymer with tunable degradation properties and an excellent safety record in diverse medical applications [12]. Preliminary investigations have suggested its feasibility in GBR [13]. To build on this, a three-dimensional (3D) preformed resorbable PLGA membrane (3D-PRPM) was developed to merge the rigidity of titanium meshes with the resorbability of collagen membranes. The aim of this study was to evaluate the clinical effectiveness and short-term safety of 3D-PRPM in localized horizontal alveolar ridge defects, with the goal of providing preliminary clinical evidence for its use as a mechanically stable resorbable barrier in GBR.

## Materials and methods

### Characterization of a 3D-PRPM

**Fig. 1:**
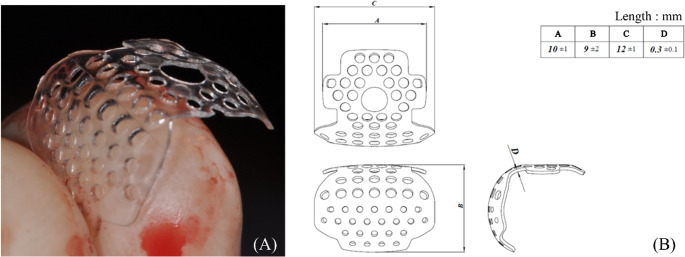
3D-preformed resorbable Polylactic-co-glycolic acid membrane (3D-PRPM). (**A**) Clinical photograph of 3D-PRPM. (**B**) Dimensional schematic showing the preformed configuration designed to maintain space for guided bone regeneration

PLGA (PURASORB PLG 8218, Corbion, The Netherlands; MW 90,000; L-lactide: glycolide ratio 82:18) was used for the fabrication of 3D-PRPM (r-Builder^®^; Osstem Implant Co., Ltd., Seoul, Korea). The polymer is manufactured under GMP standards for biomedical applications. The 3D-PRPM was fabricated by injection molding, with a standardized thickness of approximately 0.3 mm and 1.0-mm pores to facilitate vascularization and tissue integration (Fig. 1). Each membrane was preformed with dimensions sufficient to cover buccal bone defects around dental implants.

### In vitro mechanical and degradation tests

**Fig. 2:**
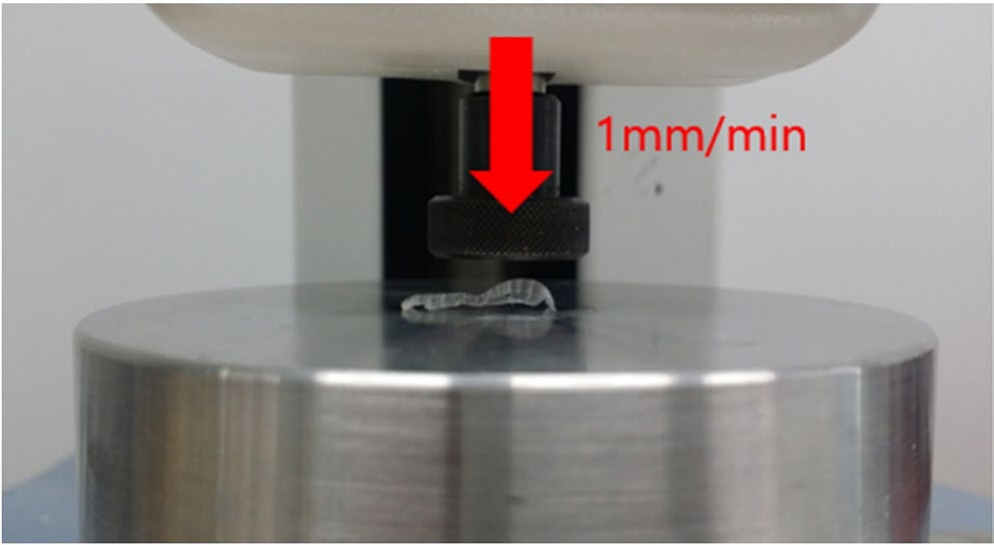
Compressive load test of 3D-preformed resorbable Polylactic-co-glycolic acid membrane

Compressive stiffness of the 3D-PRPM was evaluated using a universal testing machine, with five specimens loaded vertically at 1 mm/min until permanent deformation, and the corresponding load recorded as the critical failure point (Fig. 2). For degradation studies, specimens were incubated in Phosphate-Buffered Saline (PBS, pH 7.4) at 37 °C under gentle agitation and retrieved at 0, 2, 4, and 6 months. After blotting, all samples were air-dried at room temperature for 24 h prior to testing. Weight loss was determined gravimetrically using an analytical balance (precision ± 0.1 mg) and calculated as Weight Loss (%) = [(W₀ − Wₜ) / W₀] × 100, where W₀ represents the initial dry weight and Wₜ the dry weight at each time point. Mechanical integrity was reassessed at each interval using the same compression protocol, and pH of the degradation medium was measured in triplicate with a calibrated digital meter, using fresh solution at every time point for both 3D-PRPM and a lower molecular weight PLGA (50,000) control.

### Study design and ethical approval

This prospective, single-arm clinical trial was conducted at the department of Periodontology, Chosun University Dental Hospital, Gwangju, Republic of Korea. The study protocol was approved by the Institutional Review Board of Chosun University Dental Hospital (CUDHIRB 2405-006), and written informed consent was obtained from all participants in accordance with the Declaration of Helsinki.

### Patient selection

Twenty patients with localized alveolar ridge defects requiring implant placement with simultaneous GBR were enrolled. Inclusion criteria were: (a) ≥ 18 years of age; (b) systemically healthy status; (c) presence of localized horizontal alveolar ridge defects requiring GBR at the time of implant placement; and (d) willingness to provide written informed consent and comply with study procedures.

Exclusion criteria included: (a) systemic diseases affecting bone metabolism or wound healing; (b) heavy smoking (≥ 10 cigarettes/day); (c) alcohol or substance abuse; (d) active periodontal disease in the residual dentition; (e) pregnancy or lactation; (f) concurrent participation in another clinical trial; and (g) any condition deemed inappropriate by the investigators.

### Surgical procedure

**Fig. 3:**
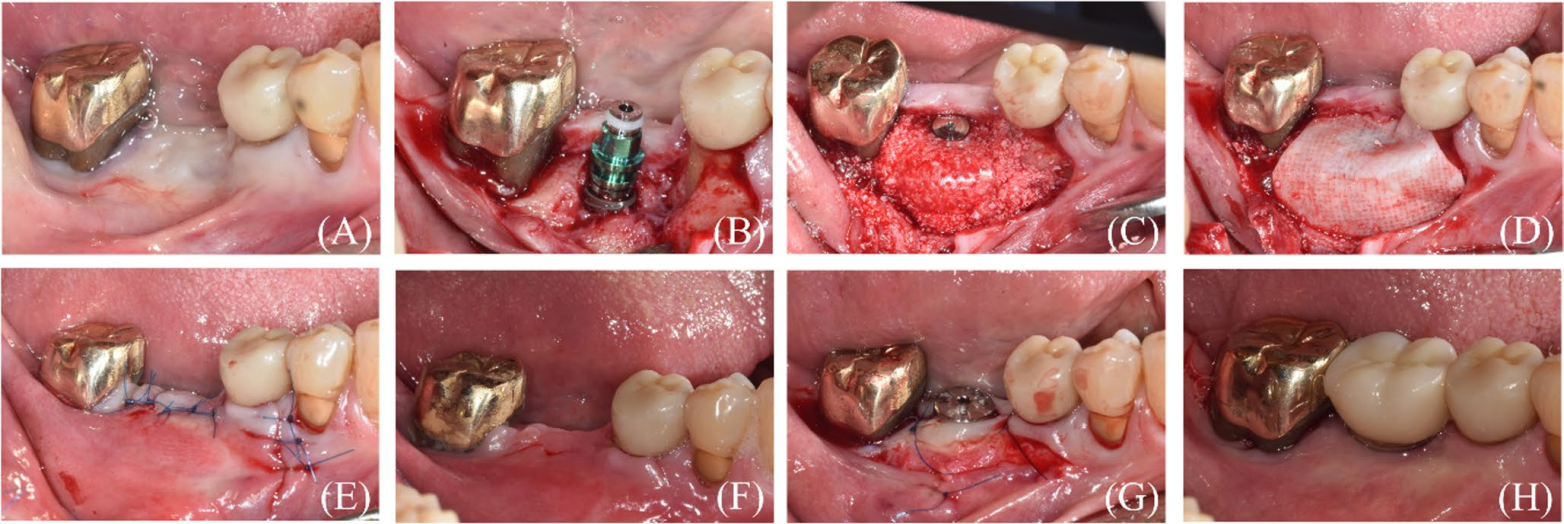
Surgical procedure of guided bone regeneration using 3D-preformed resorbable Polylactic-co-glycolic acid membrane (3D-PRPM). (**A**) Preoperative view of the edentulous site. (**B**) Implant placement following flap elevation. (**C**) Placement of xenogeneic particulate graft and coverage with 3D-PRPM. (**D**) Additional coverage with a collagen membrane. (**E**) Primary flap closure with tension-free suturing. (**F**) Clinical view before second-stage surgery. (**G**) Healing abutment connection with apically positioned flap. (**H**) Delivery of the final prosthesis

All procedures were performed by a single experienced periodontist (WPL) under local anesthesia (Fig. 3A). After a mid-crestal incision and full-thickness flap elevation, implants (TS III^®^, Osstem Implant Co., Ltd., Seoul, Korea) were placed in the planned positions (Fig. 3B). A 1-mm anchor screw was connected to the implant fixture, the peri-implant defects were filled with particulate xenogeneic bone graft material (A-Oss^®^, Osstem Implant Co., Ltd., Seoul, Korea), and the grafted sites were covered with a 3D-PRPM stabilized by the cover cap (Fig. 3C). To further reinforce the barrier function, an additional resorbable collagen membrane (OssMem soft^®^, Osstem Implant Co., Ltd., Seoul, Korea) was placed over the 3D-PRPM to enhance soft tissue adaptation and to provide an additional barrier against epithelial migration, particularly considering the pore size of the 3D-PRPM (Fig. 3D). The flaps were repositioned and sutured to achieve tension-free primary closure (Fig. 3E).

All patients received standard postoperative instructions and were prescribed analgesics (aceclofenac 100 mg, Dona-A ST, Seoul, Korea) to be taken twice daily for one week. Sutures were removed approximately 2 weeks after surgery, and patients were followed at regular intervals to assess wound healing and record any adverse events. After a 5-month healing period, second-stage surgery was performed, and final prosthetic restorations were delivered according to standard implant loading protocols.

### Radiological evaluation

CBCT scans were obtained under standardized imaging conditions (field of view diameter: 10 cm; field of view height: 5.6 cm; tube voltage: 90 kV; tube current: 8.0 mA; voxel size: 0.2 mm) by a single independent examiner (JSK) who was blinded to the surgical procedures and not involved in the clinical treatment.

**Fig. 4:**
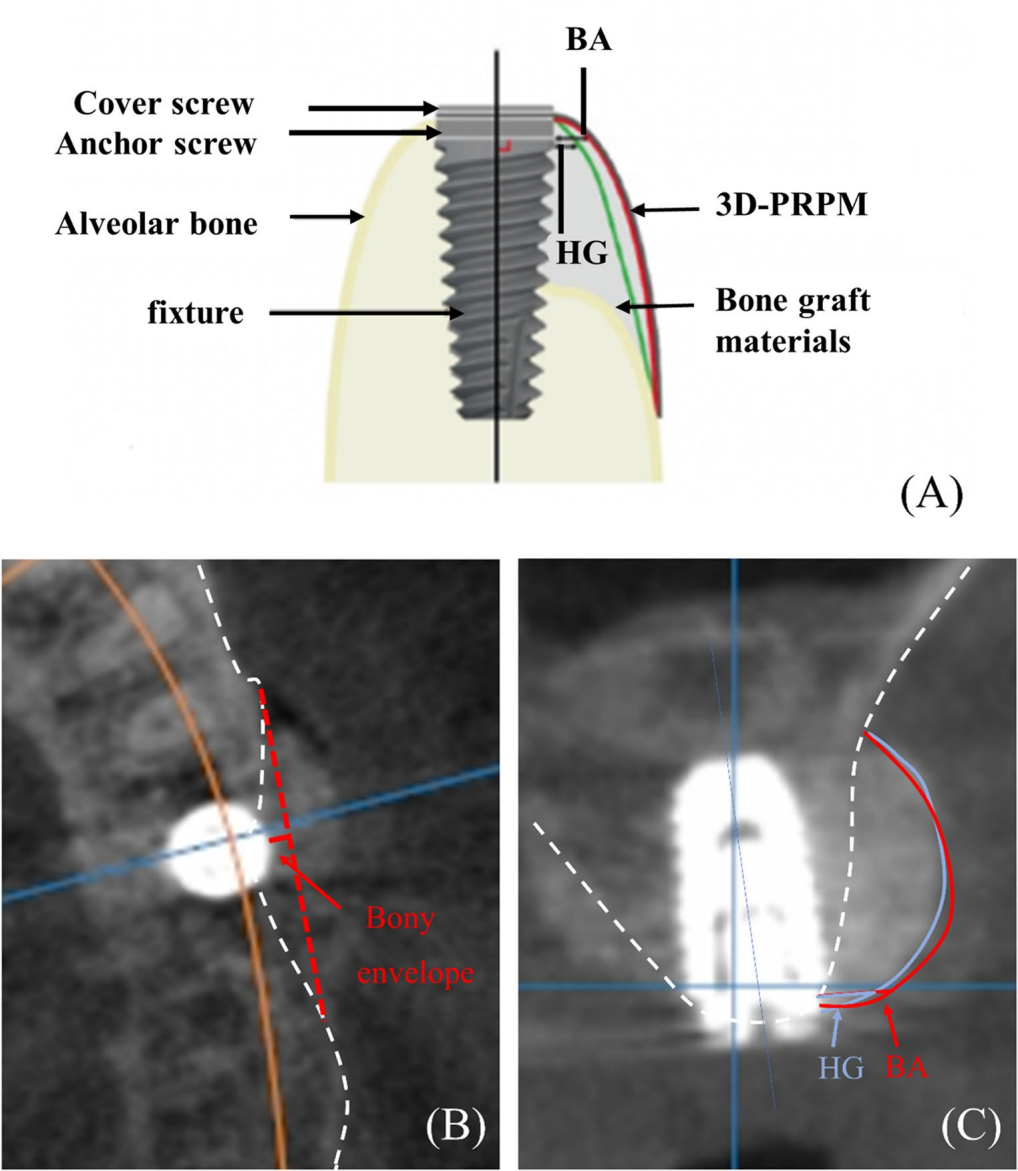
Radiographic measurements. (**A**) Schematic illustration showing bone augmentation (BA) and hard tissue gain (HG) via 3D-preformed resorbable Polylactic-co-glycolic acid membrane (3D-PRPM). (**B**) Preoperative CBCT image showing the bony envelope. (**C**) Postoperative CBCT image demonstrating BA and HG

Cross-sectional images passing through the center of each implant were used for linear measurements (Fig. 4A). A reference line perpendicular to the long axis of the implant was drawn from the platform level to the buccal aspect, and the horizontal distance from the implant surface to the outermost regenerated bone along this line was recorded (Fig. 4B). Based on these measurements, bone augmentation (BA) was defined as the horizontal thickness immediately after augmentation, and hard tissue gain (HG) as the thickness at 5 months (Fig. 4C). Bone resorption (BR) was calculated as the difference between BA and HG, and the hard tissue gain rate (HGR) as the percentage of HG relative to BA (HG/BA × 100) [4]. To evaluate the stability of regenerated hard tissue, a one-sample t-test was performed to compare the mean HGR against a reference value of 80%, which was selected based on previous clinical studies reporting mean retention rates of approximately 80–88% following GBR using titanium meshes [14–16].

The bony envelope was defined as the line connecting the most labial alveolar bone of the adjacent teeth in the 3D image. Measurements located outside the envelope were recorded as negative values, whereas those within the envelope were recorded as positive values [17]. To evaluate measurement consistency, the same examiner performed duplicate measurements at a one-week interval. The average of the two readings was adopted for subsequent analysis. Intra-examiner reliability was confirmed by intraclass correlation coefficients, which were all greater than 0.90.

### Statistical analysis

All statistical analyses were performed using SPSS software, version 27.0 (IBM Corp., Armonk, NY, USA). Descriptive statistics for continuous variables were calculated, including the mean, standard deviation (SD), minimum, maximum, and 95% confidence interval (CI). The distribution of data was assessed using the Shapiro–Wilk test.

For radiographic outcomes, BA and HG were compared using the Wilcoxon signed-rank test. HGR was expressed as the mean ± SD with 95% CI. Changes in the bony envelope across the three time points (preoperative, immediately after augmentation, and 5 months) were evaluated using the Friedman test, followed by pairwise Wilcoxon signed-rank tests with Bonferroni correction. A two-tailed p-value < 0.05 was considered statistically significant.

## Results

### Characterization of a 3D-PRPM

#### Mechanical properties of the 3D-PRPM

**Fig. 5:**
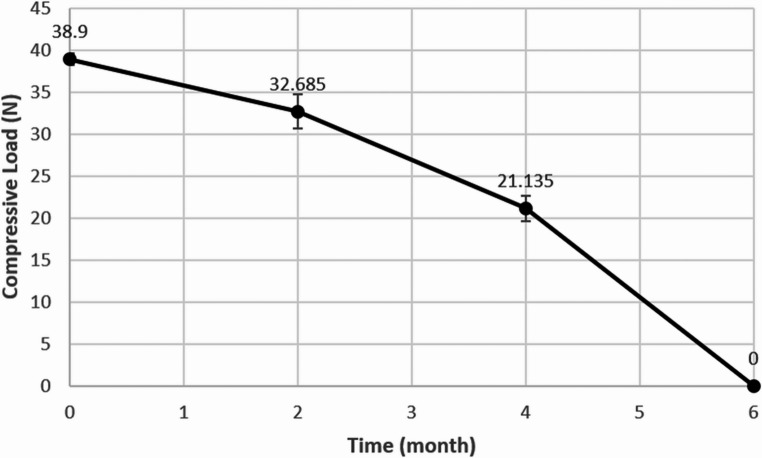
Changes in compressive load of 3D-preformed resorbable Polylactic-co-glycolic acid membrane during in vitro degradation.

At each degradation time point (0, 2, 4, and 6 months), compressive strength decreased in accordance with progressive membrane degradation. The 3D-PRPM maintained compressive loads above the 16 N threshold through 4 months, but no measurable strength was detected thereafter (Fig. 5).

#### In vitro degradation evaluation

**Fig. 6:**
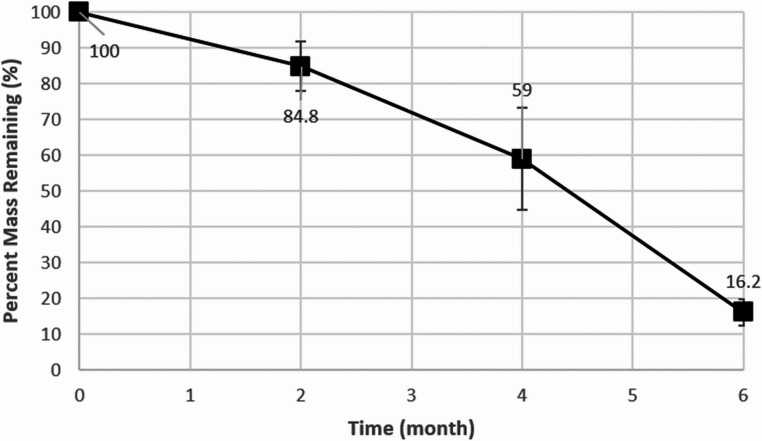
Changes in weight of 3D-preformed resorbable Polylactic-co-glycolic acid membrane during in vitro degradation

**Fig. 7:**
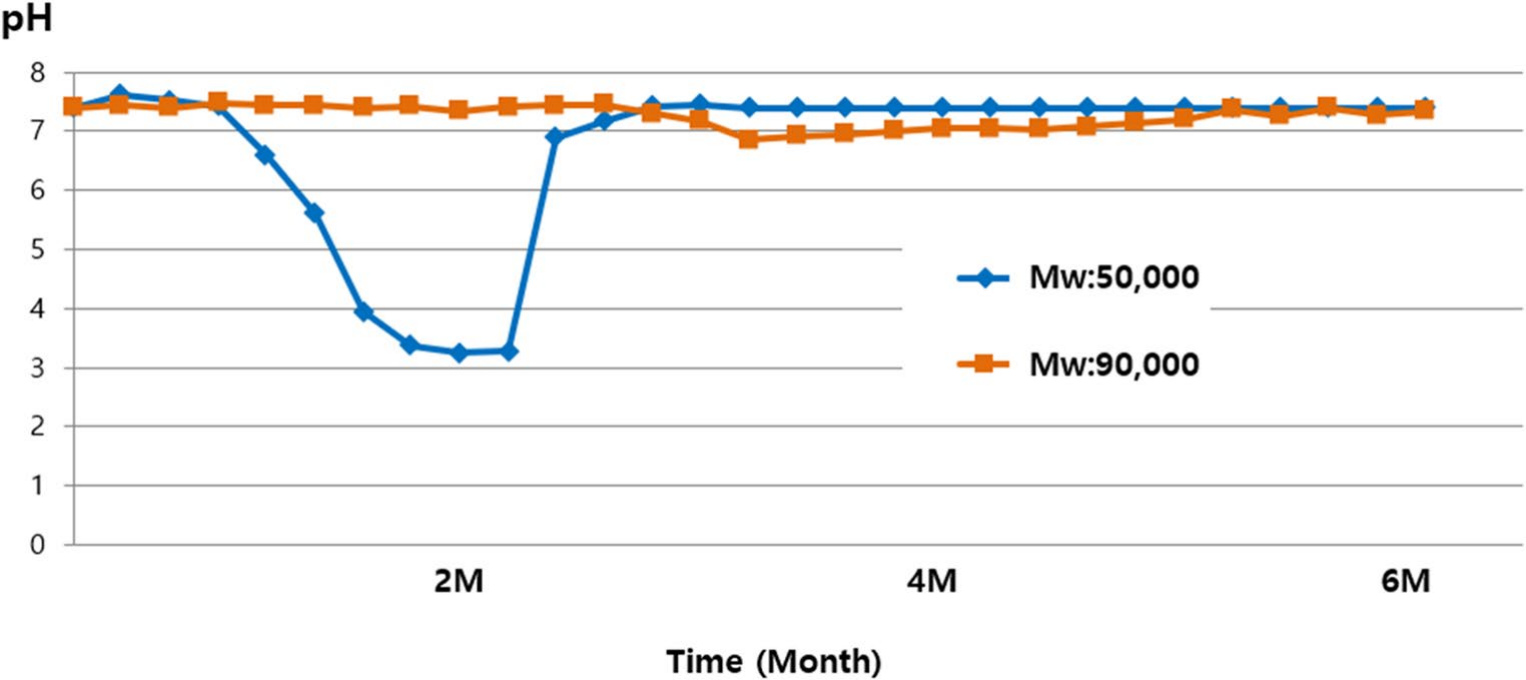
Changes in pH of 3D-preformed resorbable Polylactic-co-glycolic acid membrane during in vitro degradation

The 3D-PRPM exhibited a gradual loss of mass over time, with more than 50% of the original weight retained at 4 months in PBS, while substantial weight reduction was observed by 6 months (Fig. 6). PLGA with a molecular weight of 50,000 exhibited a marked decrease in pH due to rapid degradation and release of acidic by-products. In contrast, the 3D-PRPM composed of PLGA with a molecular weight of 90,000 showed only gradual pH changes, indicating slower degradation and reduced acidification of the medium. These findings suggest that high-molecular-weight PLGA maintains a more stable pH environment during in vitro degradation (Fig. 7).

#### Patient characteristics

A total of 20 patients with 21 implant sites were included in the final analysis. The mean age was 60.5 ± 16.6 years (range, 25–83 years), comprising 11 men (11 sites) and 9 women (10 sites) (Table 1). Defects were located in 5 anterior and 16 posterior sites. All participants successfully completed the 5-month follow-up without systemic or local complications. Notably, no cases of membrane exposure, infection, or wound dehiscence were observed during the observation period.

**Table 1:** Patient and site characteristics (*n* = 20 patients, 21 sites)

Defect site (Anterior/Posterior)	Patient no.	Age	Sex	Site location	PMH
Anterior	1	25	F	#31	N/S
	2	57	F	#41	CI
	3	54	F	#22	OPE
	4	26	M	#32	N/S
	5	54	F	#23	Angina
Posterior	6	69	M	#47	HTN
	7	29	M	#35	N/S
	8	63	M	#36	N/S
	9	66	M	#26	N/S
	10	62	M	#36	N/S
	11	81	M	#46	Angina
	12	66	M	#27	N/S
	13	65	M	#27	HTN
	14	79	M	#44	Stent
	15	83	F	#45	HTN
	16	58	M	#27	DM
	17	64	F	#35	HTN
	18	78	F	#26	Osteoporosis
	19	74	F	#14	HTN
	20	59	F	#16	Hypothyroidism
				#46	

Abbreviations: *No.* number, *PMH* past medical history, *M* male, *F* female, *N/S* no systemic disease, *HTN* hypertension, *CI* cerebral infarction, *OPE* history of gynecologic surgery, *Stent* coronary stent placement, *DM* diabetes mellitus

#### Radiographic outcomes

**Fig. 8:**
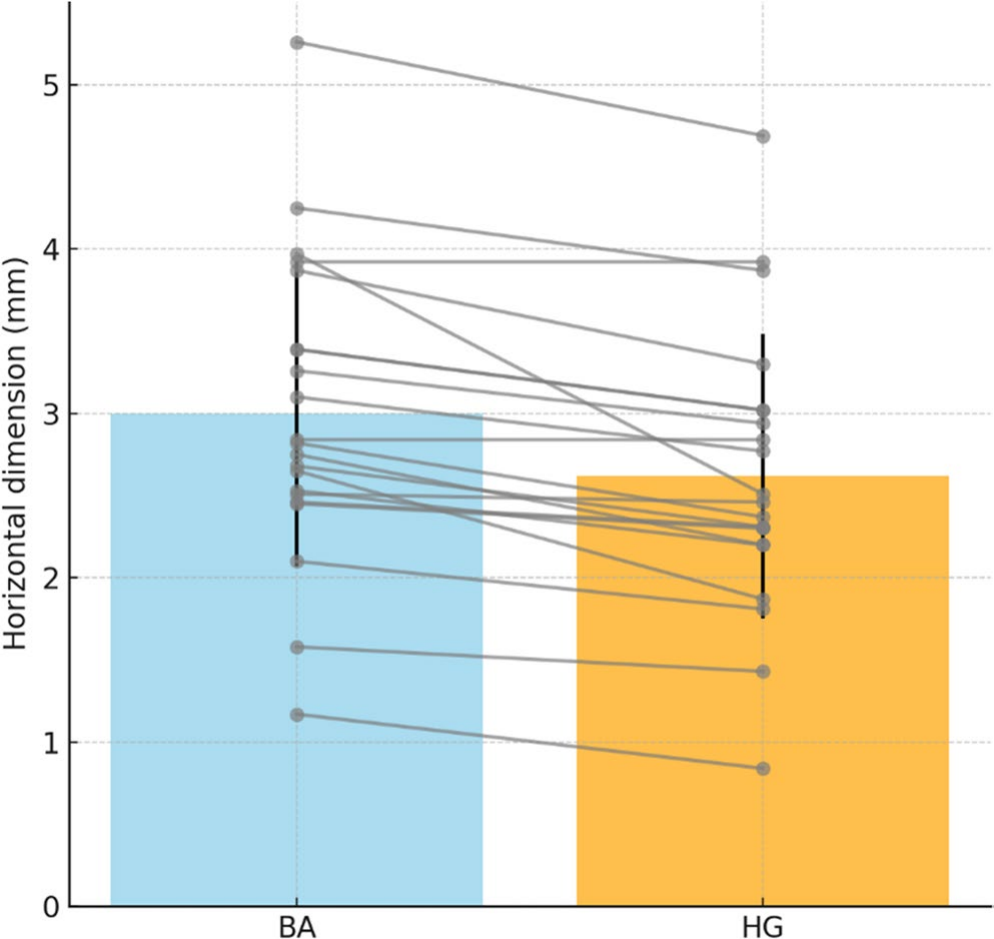
Comparison of horizontal bone augmentation (BA) and hard tissue gain (HG). Values immediately after surgery and at 5 months (*n* = 21). Each gray line represents paired values from the same site, and mean ± standard deviation (SD) values are shown as colored bars with error bars

The mean horizontal BA immediately after surgery was 2.99 ± 1.15 mm (range, 1.17–5.26 mm; median, 2.75 mm). At 5 months, the mean HG was 2.64 ± 1.10 mm (range, 0.84–4.92 mm; median, 2.37 mm). The mean BR (BA–HG) was 0.35 ± 0.23 mm (range, 0.07–1.04 mm; median, 0.36 mm). The Wilcoxon signed-rank test confirmed that BA was significantly greater than HG (Z = − 4.016, *p* < 0.001), with a large effect size (*r* = 0.88). The Hodges–Lehmann median difference was 0.35 mm (95% CI, 0.21–0.50), indicating partial resorption during healing while maintaining substantial horizontal volume (Table 2; Fig. 8).

**Table 2:** Radiographic outcomes (*n* = 21)

Variables	Values		
	Mean ± SD	Median	Range
BA (mm)	2.99 ± 1.15	2.75	1.17–5.26
HG (mm)	2.64 ± 1.10	2.37	0.84–4.92
BR (BA–HG) (mm)	0.35 ± 0.23	0.36	0.07–1.04
HGR (%)	87.2 ± 8.2	89.1	71.2–97.4

Abbreviations: *BA* bone augmentation, *HG* hard tissue gain, *BR* bone resorption, *HGR* hard tissue gain rate, *SD* standard deviation

#### Hard tissue gain rate

**Fig. 9:**
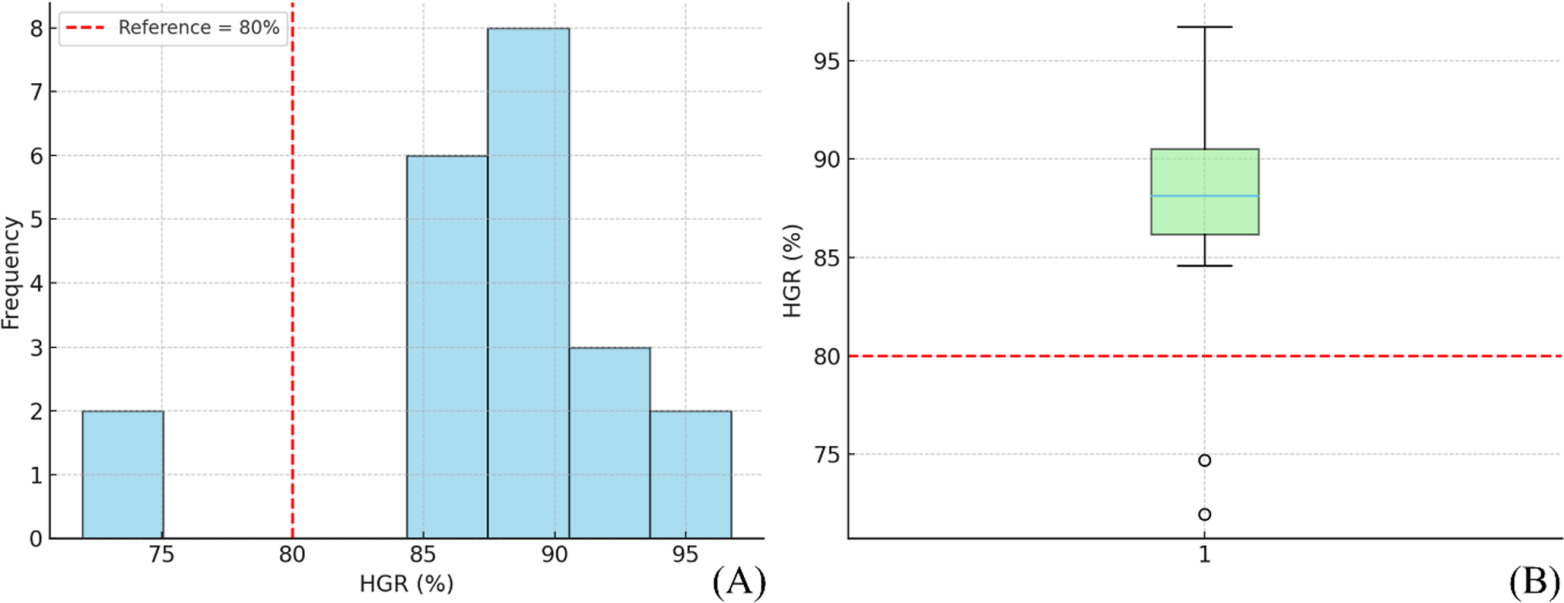
Distribution of hard tissue gain rate (HGR). (**A**) Histogram showing clustering between 70% and 100%. (**B**) Box plot showing a median of 89.09% with an interquartile range (IQR) of 85–91%

The mean HGR was 87.2% ± 8.2% (95% CI, 83.4–90.8%; median, 89.09%; range, 71.2–97.4%). A one-sample t-test against the reference value of 80% demonstrated that HGR was significantly higher than the threshold (t = 3.99, *p* < 0.001, Cohen’s d = 0.87, Hedges’ g = 0.84). The mean difference from the reference value was 7.2% (95% CI, 3.4–10.9). Most implant sites clustered above 85%, with only a few outliers, indicating consistently stable outcomes across the study population. Values are presented as mean ± SD, median, and range (Table 2; Fig. 9).

#### Changes in the bony envelope

**Fig. 10:**
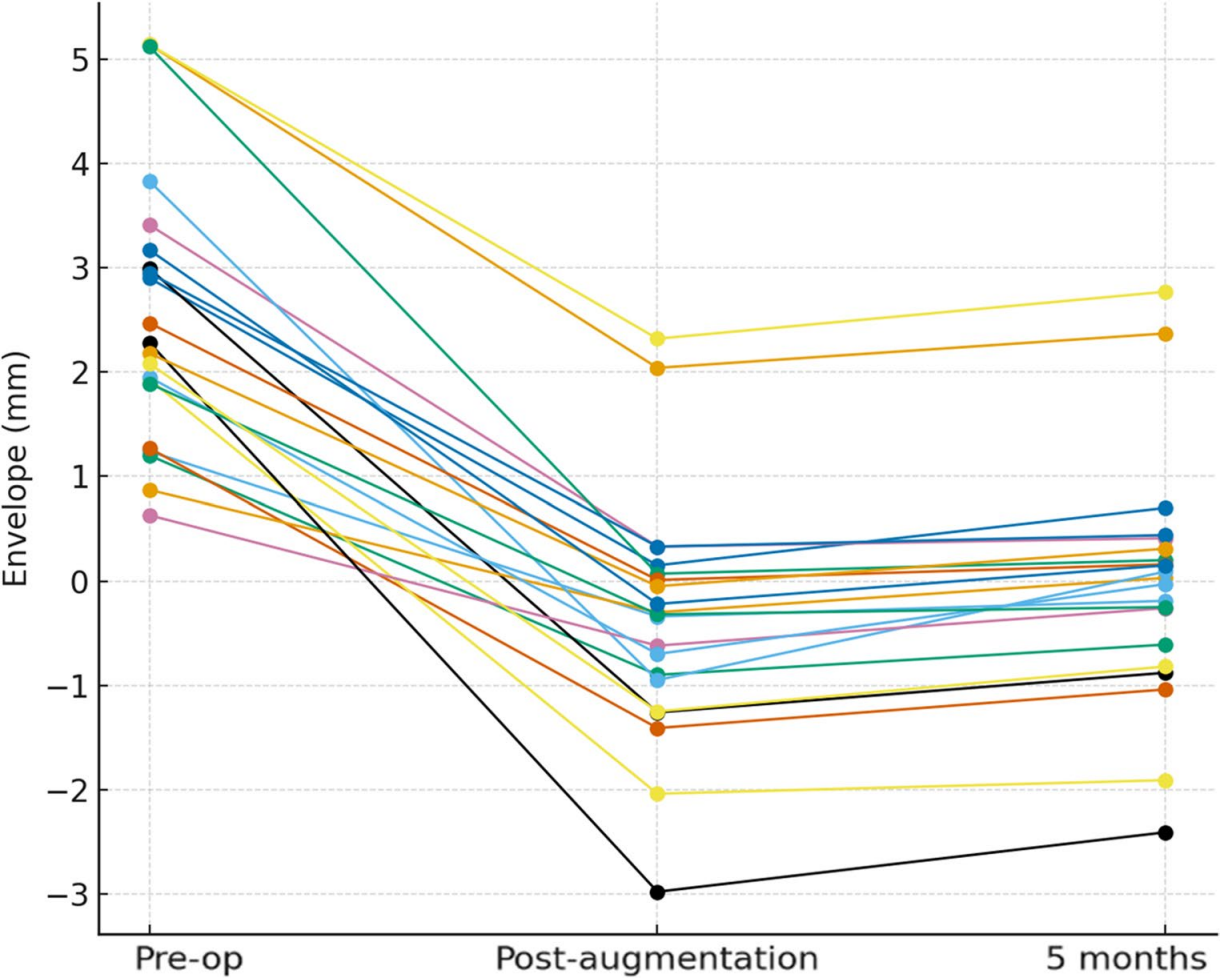
Changes in horizontal distance from the bony envelope. Individual changes in horizontal distance from the bony envelope at baseline, post-augmentation, and 5 months. Positive values indicate positions within the bony envelope, and negative values indicate those beyond it

The distance from the bony envelope to the regenerated hard tissue showed significant temporal changes according to the Friedman test (*p* < 0.001). The mean values were − 0.39 ± 1.18 mm immediately after augmentation and − 0.04 ± 1.16 mm at 5 months, suggesting that the regenerated contour was generally maintained slightly beyond the original bony envelope. All sites were located within the bony envelope at baseline; however, 14 sites (66.7%) extended beyond the envelope immediately after augmentation, and 10 of these (71.4%) continued to exhibit negative values after 5 months, indicating that most of the augmented contours remained beyond the original alveolar envelope despite partial resorption (Table 3; Fig. 10).

**Table 3:** Distance from bony envelope to hard tissue profile (*n* = 21)

Time point	Mean ± SD (mm)	Median (mm)	Range (mm)
Baseline	2.60 ± 1.35	2.28	0.63–5.14
Post-augmentation	–0.39 ± 1.18	–0.32	–2.98–2.32
5 months	–0.04 ± 1.16	0.03	–2.41–2.77

Abbreviations: *SD* standard deviation. Positive values indicate positions within the bony envelope, and negative values indicate those beyond it

## Discussion

**Fig. 11:**
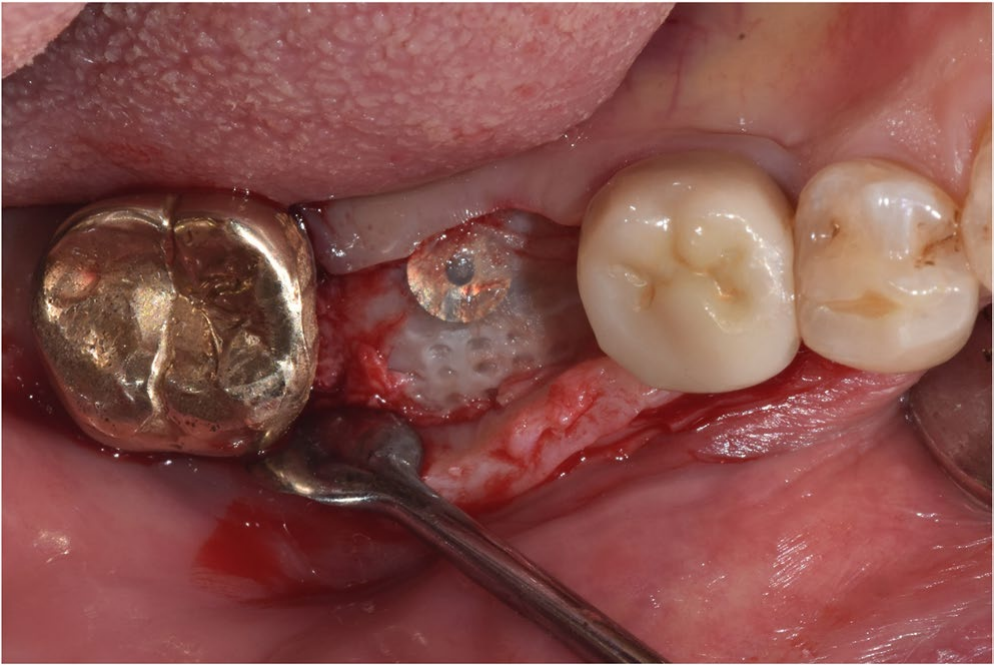
Clinical view at 5 months. Intentional re-entry at a representative site shows that the 3D-preformed resorbable Polylactic-co-glycolic acid membrane remained stable without evidence of exposure, infection, or displacement

To the best of our knowledge, this is the first clinical study to evaluate the clinical effectiveness and short-term safety of guided bone regeneration using 3D-PRPM in conjunction with simultaneous implant placement. The study included 20 patients (11 men and 9 women), with a total of 21 implant sites comprising 5 anterior and 16 posterior defects. The mean horizontal augmentation was approximately 3 mm, and the 5-month hard tissue gain was 2.64 mm, corresponding to an HGR of about 87%. Importantly, no cases of membrane exposure, infection, or wound dehiscence were observed throughout the observation period. Favorable outcomes were consistently achieved in both anterior and posterior regions, suggesting that 3D-PRPM is effective across various anatomical sites. Clinical re-entry during second-stage surgery confirmed that the augmented bone volume was well maintained (Fig. 11), aligning with radiographic findings and highlighting the excellent space-maintaining capacity of the 3D-PRPM. These results support its ability to provide stable bone regeneration without the complications commonly associated with non-resorbable membranes.

Previous studies have reported horizontal bone augmentation of approximately 2–4 mm using titanium meshes, with volumetric retention rates around 78.6% and predictable regenerative outcomes [18, 19]. In contrast, the present study achieved a mean HG of 2.64 ± 1.10 mm and a mean HGR of 87.2 ± 8.2%, which are comparable to or slightly higher than the outcomes reported for titanium meshes in previous investigations. Specifically, a mean horizontal gain of 2.9 mm with an HGR of 83% was reported in a recent clinical study [4], and 3.1 mm with an HGR of 82% was observed in another retrospective analysis [14], while systematic reviews have summarized mean horizontal gains of approximately 3–4 mm [5, 18].

 However, exposure remains a major limitation, occurring in 15–25% of cases and up to 40–50% in certain series, often resulting in infection, graft loss, and the need for secondary removal, which increases patient morbidity and surgical complexity [5, 7]. Similarly, 3D-preformed titanium meshes have yielded comparable horizontal bone gains (approximately 2–3 mm) and volume retention rates of around 80%, although exposure rates ranging from 5% to 12% continue to be reported [4, 14]. In the present study, 3D-PRPM demonstrated favourable horizontal augmentation and volume retention without membrane exposure or the need for secondary removal. These findings suggest that the membrane may help reduce certain clinical drawbacks commonly associated with non-resorbable barriers, although direct comparative conclusions cannot be drawn because of the single-arm study design.

Collagen membranes are widely used in GBR because of their excellent biocompatibility and clinical manageability [10]. However, their insufficient rigidity often leads to membrane collapse in large or non-contained defects, compromising space maintenance and regenerative outcomes, even when fixation devices are used [20, 21]. In contrast, 3D-PRPM retains the favourable biocompatibility and degradability of PLGA while providing enhanced mechanical stability and an anatomically preformed design that may help preserve graft volume during healing [22–24]. In the present protocol, the additional collagen membrane was used not only to improve soft tissue adaptation but also to provide an additional superficial barrier against epithelial downgrowth over the porous structure of the 3D-PRPM. These complementary mechanical and biological characteristics likely contributed to the favorable volumetric stability and high retention rate observed in this study. Previous envelope-based analyses have shown that when GBR is performed using collagen membranes, the initially overbuilt contour tends to regress within the bony envelope during healing [17], whereas augmentation with preformed titanium meshes generally maintains the regenerated ridge contour outside the bony envelope [4]. In the present study, 3D-PRPM exhibited a comparable pattern of stability; the mean distance from the bony envelope to the regenerated hard tissue was − 0.39 ± 1.18 mm immediately after augmentation and remained at − 0.04 ± 1.16 mm after 5 months, indicating that the augmented contour was preserved slightly beyond the original bone envelope.

 Beyond mechanical advantages, 3D-PRPM also offers several clinical benefits. It enables simultaneous implant placement with GBR, reducing treatment time compared with staged protocols, and eliminates the need for fixation devices, simplifying the surgical procedure. As a resorbable barrier, it obviates secondary removal and improves patient comfort. These results are consistent with previous clinical and experimental evidence, where dimensional stability of 3D-PRPM has been verified in clinical applications [13], and PLGA-based composites have demonstrated improved osteoconductive properties in preclinical studies [25, 26]. Collectively, these findings suggest that 3D-PRPM may serve as a clinically effective resorbable barrier for horizontal ridge augmentation, demonstrating favorable stability and handling characteristics in the present study. Furthermore, future developments may enable the fabrication of patient-specific membranes using additive manufacturing technologies, potentially expanding the clinical applicability of this material. Although the present study focused on horizontal augmentation, the mechanical properties of the 3D-PRPM suggest potential applicability in vertical augmentation, which warrants further investigation.

 Despite these favorable outcomes, potential limitations of PLGA membranes should be acknowledged. During degradation, PLGA generates lactic and glycolic acids, which can reduce local pH and potentially induce inflammatory responses or tissue alterations [23, 24]. Such effects were not observed in the present study, but further investigation is needed, particularly in large defects or in systemically compromised patients. The present findings, supported by in vitro data, indicate that the high-molecular-weight PLGA used in the 3D-PRPM degrades gradually with minimal pH fluctuation, while the preformed configuration provides effective space maintenance. In addition, the degradation profile of the 3D-PRPM, which maintained compressive strength above the critical threshold (16 N) for up to 4 months, appears to align well with the biological timeline required for bone regeneration, particularly during the early phase of graft stabilization. These findings suggest that appropriately engineered PLGA membranes may serve as clinically effective resorbable barriers in GBR.

This study has several limitations. First, the sample size was relatively small, and the absence of a control group limits direct comparison with other treatment modalities. In particular, because the 3D-PRPM was used in combination with an additional collagen membrane, and neither a collagen membrane-only group nor a 3D-PRPM-only group was included, direct comparative conclusions between membrane types cannot be drawn. Additionally, the follow-up period was restricted to 5 months, precluding assessment of long-term bone stability, remodeling, or implant survival. Furthermore, histological evaluation was not performed, and therefore the extent of true new bone formation could not be directly verified. Within these limitations, the present findings suggest that 3D-PRPM may serve as a clinically effective resorbable barrier for horizontal ridge augmentation, demonstrating favorable volumetric stability and clinical handling without the need for secondary removal. However, future randomized controlled trials with larger sample sizes, longer follow-up periods, and direct histological assessment are required to further validate these findings and support clinical decision-making in alveolar ridge augmentation.

## Conclusion

Within the limitations of this prospective single-arm study, GBR using 3D-PRPM demonstrated predictable horizontal ridge augmentation with high volumetric stability and an absence of major complications. The membrane provided stable space maintenance without the need for fixation or secondary removal, resulting in consistent clinical outcomes. These findings suggest that 3D-PRPM may serve as a clinically effective resorbable barrier for horizontal ridge augmentation. Further controlled studies with larger cohorts, longer follow-up periods, and histological validation are warranted to confirm its long-term regenerative potential and biological safety.

## Data Availability

The datasets generated and analyzed during the current study are not publicly available due to privacy concerns and ethical restrictions related to participant information. However, de-identified data may be made available by the corresponding author upon reasonable request and with approval from the Institutional Review Board.
